# Impact of analytical and biological variations on classification of diabetes using fasting plasma glucose, oral glucose tolerance test and HbA1c

**DOI:** 10.1038/s41598-017-14172-8

**Published:** 2017-10-20

**Authors:** Jia Hui Chai, Stefan Ma, Derick Heng, Joanne Yoong, Wei-Yen Lim, Sue-Anne Toh, Tze Ping Loh

**Affiliations:** 1 Saw Swee Hock School of Public Health, National University of Singapore, Singapore, Singapore; 2 Epidemiology & Disease Control Division, Ministry of Health, Singapore, Singapore; 3 Public Health Group, Ministry of Health, Singapore, Singapore; 4 Research and Development Office, Agency for Integrated Care, Singapore, Singapore; 5 Department of Medicine, National University Hospital, Singapore, Singapore; 6 Department Laboratory Medicine, National University Hospital, Singapore, Singapore; 7 Biomedical Institute for Global Health Research and Technology, National University of Singapore, Singapore, Singapore

## Abstract

Historically, diabetes is diagnosed by measuring fasting (FPG) and two-hour post oral glucose load (OGTT) plasma concentration and interpreting it against recommended clinical thresholds of the patient. More recently, glycated haemoglobin A1c (HbA1c) has been included as a diagnostic criterion. Within-individual biological variation (CVi), analytical variation (CVa) and analytical bias of a test can impact on the accuracy and reproducibility of the classification of a disease. A test with large biological and analytical variation increases the likelihood of erroneous classification of the underlying disease state of a patient. Through numerical simulations based on the laboratory results generated from a large population health survey, we examined the impact of CVi, CVa and bias on the classification of diabetes using fasting plasma glucose (FPG), oral glucose tolerance test (OGTT) and HbA1c. From the results of the simulations, HbA1c has comparable performance to FPG and is better than OGTT in classifying subjects with diabetes, particularly when laboratory methods with smaller CVa are used. The use of the average of the results of the repeat laboratory tests has the effect of ameliorating the combined (analytical and biological) variation. The averaged result improves the consistency of the disease classification.

## Introduction

Diabetes mellitus is a chronic disease characterized by impaired glucose metabolism. The hallmark of this disease is persistent elevation of glucose concentration in the blood^1^. Historically, diabetes is diagnosed by measuring plasma glucose concentration and interpreting it against recommended clinical thresholds, which are dependent on the state of satiety (fasting, post-oral glucose load or random) of the patient. More recently, glycated haemoglobin A1c (HbA1c) has been included as a diagnostic criterion by several professional bodies, including the American Diabetes Association and World Health Organisation^1,2^.

The advantages and disadvantages of the different diagnostic test have been well discussed^3,4^. These discussions tend to focus on the clinical performance or operational convenience of the tests without explicit consideration for the impact of analytical and biological variation on the classification of diabetes. Yet, biological and analytical variations of a test can have a large impact on the accuracy and reproducibility of the classification of a disease. A test with large biological and analytical variation increases the probability of the result of a patient falling further from his true homeostatic set point. This increases the likelihood of erroneous classification of the underlying disease state of a patient^5^. Indeed, it has been reported that only 70% of patients diagnosed with diabetes using oral glucose tolerance test were still classified as having diabetes when the test was repeated three weeks later^3^.

Accurate classification of patients is important for optimal clinical care. It also improves the selection of patients in clinical studies and sharpens the distinction between disease and control groups^5^. This in turn enhances the observed difference between disease and control groups. Occasionally, repeat testing is performed to resolve discrepant disease classification by different laboratory tests. However, there is currently a lack of consensus on how best to interpret the replicate results.

Through numerical simulations based on the laboratory results generated from a large population health survey, we examined (1) the impact of biological and analytical variations and between-laboratory method bias on the classification of diabetes using fasting plasma glucose (FPG), oral glucose tolerance test (OGTT) and HbA1c, and (2) the best strategy to interpret limited repeat blood testing to reduce the variation in result to achieve more accurate disease classification.

## Methods

### Study subjects

This study used data from the cross-sectional National Health Survey (Singapore) collected between 17 March 2010 and 13 June 2010. All participants provided written informed consent for further analysis of the collected data. This study design received ethics board approval (Medical & Dental Board, Health Promotion Board, ref: 005/2009) and its details are available in the official report. This study only involves statistical analysis of data previously collected from the survey and the methods described in this study were performed according to relevant local guidelines and regulation.

Briefly, the survey employed a two-phase sampling strategy. In the first phase, the geographical zones and residential dwelling units were stratified and selected to yield a representative dwelling type distribution. In the second phase, 7,696 individuals were randomly sampled from households identified in phase 1 and invited to participate in the survey. Ethnic minorities were over-sampled to achieve an ethnic composition of 30% Chinese, 30% Malays, 30% Indians and 10% others.

Of the 7,512 eligible individuals aged 18 to 79 years, 4337 participated in the survey (representing a participation rate of 57.7%). Only subjects without prior history of diabetes were included in the final analysis.

### Blood tests

Blood samples from participants were collected after an overnight fasting of at least ten hours, using standard phlebotomy procedure. The OGTT was performed by orally administering 75 g of glucose (Trutol), and measurement of the plasma glucose concentration was repeated two hours later.

### World Health Organisation definition of glycaemic status

Normal FPG was defined as <6.1 mmol/L while impaired FPG was defined as 6.1 mmol/L to 6.9 mmol/L. Normal OGTT was defined as <7.8 mmol/L and impaired OGTT was defined as 7.8 mmol/L to 11.0 mmol/L. Normal HbA1c was defined as <6% while pre-diabetes was defined as 6% to 6.4%. Diabetes is defined as having any of the following: FPG ≥7.0 mmol/L, OGTT ≥11.1 mmol/L or HbA1c ≥6.5%.

### American Diabetes Association definition of glycaemic status

Normal FPG was defined as <5.6 mmol/L while impaired FPG was defined as 5.6 mmol/L to 6.9 mmol/L. Normal OGTT was defined as <7.8 mmol/L and impaired OGTT was defined as 7.8 mmol/L to 11.0 mmol/L. Normal HbA1c was defined as <5.7% while pre-diabetes was defined as 5.7% to 6.4%. Diabetes was defined as having any of the following: FPG ≥7.0 mmol/L, OGTT ≥11.1 mmol/L or HbA1c ≥6.5%.

### Analytical variation and bias data

The data for analytical performance were extracted from external quality assurance programs and the literature^3,6^. The analytical variation was defined as the analytical coefficient of variation (CVa) of the laboratory tests as reported in the external quality assurance programs and literature. The CVa for the plasma glucose and HbA1c measurements were 2.5% and 3.5%, respectively. On the other hand, between-laboratory bias for plasma glucose and HbA1c measurements were −6% to +7%% and −3% to +2.5%, respectively. For HbA1c, an additional level of CVa of 2% that could be achieved by certain laboratory methods was also examined. The CVa for plasma glucose is the same for FPG and OGTT since they share the same laboratory method.

### Biological variation data

The within-person biological variation (CVi) data was obtained from the database curated by Ricos and her colleagues. The CVi for FPG, OGTT and HbA1c were 5.7%, 16.7% and 1.8%, respectively. The biological variation for plasma glucose and HbA1c were assumed to be similar in healthy subjects and patients with diabetes, as demonstrated previously^7^.

### Statistical analysis

For the purpose of this study, the original laboratory results of the subjects derived from the National Health Survey were considered as the homeostatic set point (i.e. ‘true values’) of the subjects. They were used to assign the disease classification of the subjects according to the diagnostic criteria above.

To examine the reproducibility of the disease classification for each biochemistry test for diabetes, 10,000 random results were generated from a normal distribution, which incorporated the CVa and CVi, around the true value of each subject. This had the effect of simulating repeat testing of an individual patient. Each of the randomly generated results was then classified according to the diagnostic criteria above. The percentages of the simulated results falling into different disease classifications for all the subjects were compared against the disease classification using the ‘true value’ of each subject and summarized.

In clinical practice or research setting, repeat testing is sometimes undertaken to resolve discrepant classification based on different laboratory test. Generally, the tests are repeated not more than three times due to operational, financial and ethical constraints. To examine the best strategy to interpret these replicate results, two and three random results were generated for each subject as described above. The average value of the simulated results was then used to determine the disease classification for each of the subjects.

Furthermore, a ‘best-of-two’ interpretation, where any two concordant disease classification produced by the three simulated results was taken as the final classification for the subject, was also examined. The above exercises were simulated for 10,000 rounds for each subject. The percentages of the simulated results falling into different disease classifications for all the subjects were compared against the disease classification using the ‘true value’ of each subject and summarized.

### Data availability

The data contained in this submission belongs to the Ministry of Health and is not available for public access under local regulations. Interested party can contact Dr. Stefan Ma (Stefan_MA@moh.gov.sg).

## Results

In total 3326 subjects without prior history of diabetes were included in this study. The demographic characteristics of the participants are summarised in Table 1. The distribution of the FPG, OGTT and HbA1c results of these subjects and the dispersion (as represented by 95% confidence intervals) of the results around the WHO diagnostic thresholds are shown in Supplemental Figs 1–3. The density plots of the laboratory results by race are shown in Supplemental Figs 4–6. The correlation among the three laboratory tests (FPG, OGTT and HbA1c) in the study population is provided in Supplemental Table 1.

**Table 1:** Demographic characteristics of participants included in this study.

**N** = **3326**	**Frequency/Mean**	**Percent (%)/SD**
**Ethnicity**		
Chinese	1097	33
Malay	950	29
Indian	1014	30
Others	265	8
**Gender**		
Male	1589	48
Female	1737	52
**Age (years)**		
19 and below	105	3
20–24	268	8
25–29	295	9
30–34	370	11
35–39	446	13
40–44	449	14
45–49	446	13
50–54	333	10
55–59	231	7
60–64	147	5
65 and above	236	7
Body Mass Index (BMI)*		
Underweight	164	5
Normal	1432	43
Overweight	1197	36
Obese	531	16
**Laboratory test**		
Fasting Plasma Glucose	5.4 mmol/L	1.2
OGTT	7 mmol/L	3.2
HbA1c	5.8%	0.7

*2 subjects did not have their BMI recorded in the survey.

Overall, FPG had the most consistent classification of subjects with diabetes, and was followed by HbA1c, whose performance improved and became comparable to FPG when a smaller CVa of 2% was considered (Tables 2 and 3). On the other hand, FPG and OGTT were most consistent in classifying normal subjects. HbA1c was most consistent in classifying subjects with pre-diabetes. The presence of positive bias improved the consistency of the classification of subjects with diabetes but worsened the consistency of classification of normal subjects. By contrast, the presence of negative bias improved the consistency of classification of normal subjects.

**Table 2:** Proportion of patients who are misclassified when laboratory testing for diabetes is repeated (World Health Organisation criteria).

Tests	True classification	Classification after repeated tests	Without bias (%)	With positive bias (%)	With negative bias (%)
Fasting Plasma Glucose Biological variation: 5.7% Analytical variation: 2.5%	Normal (<6.1 mmol/L)	Normal	96.8	87.1	99.5
		Impaired	3.2	12.6	0.5
		Diabetes	0	0.3	0
	Impaired (6.1–6.9 mmol/L)	Normal	27.2	6.3	59.7
		Impaired	63	57.6	38.8
		Diabetes	9.8	36.1	1.5
	Diabetes (≥7 mmol/L)	Normal	0.3	0	2
		Impaired	10.2	2.4	22.2
		Diabetes	89.5	97.6	75.8
OGTT Biological variation: 16.7% Analytical variation: 2.5%	Normal (<7.8 mmol/L)	Normal	91.5	85.5	95.4
		Impaired	8.5	14.4	4.6
		Diabetes	0	0.1	0
	Impaired (7.8–11.0 mmol/L)	Normal	26.9	17.4	37.8
		Impaired	61.7	62.7	56.1
		Diabetes	11.4	19.9	6.1
	Diabetes (≥11.1 mmol/L)	Normal	1	0.5	1.8
		Impaired	15.9	10.3	22.1
		Diabetes	83.1	89.2	76.1
HbA1c Biological variation: 1.8% Analytical variation: 2%	Normal (<6%)	Normal	93.2	80.6	99.1
		Pre-diabetes	6.8	19.3	0.9
		Diabetes	0	0.1	0
	Pre-diabetes (6–6.4%)	Normal	23.4	6.9	56.7
		Pre-diabetes	70.3	73.6	42.5
		Diabetes	6.3	19.6	0.8
	Diabetes (≥6.5%)	Normal	0	0	0.5
		Pre-diabetes	10.3	3	24.6
		Diabetes	89.7	97	74.9
HbA1c Biological variation: 1.8% Analytical variation: 3.5%	Normal (<6%)	Normal	89.2	77.3	97
		Pre-diabetes	10.7	22	3
		Diabetes	0.1	0.7	0
	Pre-diabetes (6–6.4%)	Normal	28.8	13.4	54.7
		Pre-diabetes	60	61.5	42.4
		Diabetes	11.2	25.1	2.9
	Diabetes (≥6.5%)	Normal	0.4	0.1	2.1
		Pre-diabetes	12.5	5.8	22.9
		Diabetes	87.1	94.1	75

A between-laboratory positive bias of +7% and +2.5% were introduced for the fasting plasma glucose/oral glucose tolerance test, and HbA1c, respectively. A between-laboratory negative bias of −6% and −3% were introduced for the fasting plasma glucose/oral glucose tolerance test, and HbA1c, respectively.

**Table 3:** Proportion of patients who are misclassified when laboratory testing for diabetes is repeated (American Diabetes Association criteria).

Tests	True classification	Classification after repeated tests	Without bias (%)	With positive bias (%)	With negative bias (%)
Fasting Plasma Glucose Biological variation: 5.7% Analytical variation: 2.5%	Normal (<5.6 mmol/L)	Normal	91.5	70.5	98.7
		Impaired	8.5	29.4	1.3
		Diabetes	0	0	0
	Impaired (5.6–6.9 mmol/L)	Normal	25.3	6.1	54.3
		Impaired	71.6	81.3	45.3
		Diabetes	3.1	12.6	0.5
	Diabetes (≥7 mmol/L)	Normal	0	0	0.1
		Impaired	10.5	2.4	24.1
		Diabetes	89.5	97.6	75.8
OGTT Biological variation: 16.7% Analytical variation: 2.5%	Normal (<7.8 mmol/L)	Normal	91.5	85.5	95.4
		Impaired	8.5	14.4	4.6
		Diabetes	0	0.1	0
	Impaired (7.8–11.0 mmol/L)	Normal	26.9	17.4	37.8
		Impaired	61.7	62.7	56.1
		Diabetes	11.4	19.9	6.1
	Diabetes (≥11.1 mmol/L)	Normal	1	0.5	1.8
		Impaired	15.9	10.3	22.1
		Diabetes	83.1	89.2	76.1
HbA1c Biological variation: 1.8% Analytical variation: 2%	Normal (<5.7%)	Normal	86.8	66.3	98.1
		Pre-diabetes	13.2	33.7	1.9
		Diabetes	0	0	0
	Pre-diabetes (5.7–6.4%)	Normal	17.2	4.9	44.3
		Pre-diabetes	80.6	88.3	55.4
		Diabetes	2.2	6.8	0.3
	Diabetes (≥6.5%)	Normal	0	0	0
		Pre-diabetes	10.3	3	25.1
		Diabetes	89.7	97	74.9
HbA1c Biological variation: 1.8% Analytical variation: 3.5%	Normal (<5.7%)	Normal	81.3	64.2	94.3
		Pre-diabetes	18.7	35.8	5.7
		Diabetes	0	0	0
	Pre-diabetes (5.7–6.4%)	Normal	22.3	10.1	44.4
		Pre-diabetes	73.7	80.2	54.6
		Diabetes	4	9.7	1
	Diabetes (≥6.5%)	Normal	0	0	0.1
		Pre-diabetes	12.9	5.9	24.9
		Diabetes	87.1	94.1	75

A between-laboratory positive bias of +7% and +2.5% were introduced for the fasting plasma glucose/oral glucose tolerance test, and HbA1c, respectively. A between-laboratory negative bias of −6% and −3% were introduced for the fasting plasma glucose/oral glucose tolerance test, and HbA1c, respectively.

The potential impact of such misclassifications on disease prevalence can be assessed by simply dividing the number of misclassified subjects with the original number of subjects within each diagnostic category (Table 4). The prevalence of impaired glycaemia/pre-diabetes categories were most affected by the misclassified subjects due to a combination of a relatively high number of normal subjects being misclassified as being impaired glycaemia/pre-diabetes and a relatively low number of subjects who were originally classified in that category.

**Table 4:** Impact of the subject misclassification secondary to analytical and biological variation alone on the potential increase in the prevalence of various diagnostic categories.

Potential increase in prevalence caused by misclassified subjects						
Original classification	WHO criteria			ADA criteria		
	Normal (%)	Impaired (%)	Diabetes (%)	Normal (%)	Impaired (%)	Diabetes (%)
Normal fasting plasma glucose	—	46.8	0	—	32.5	0
Impaired fasting plasma glucose	1.9	—	14.4	6.6	—	14.2
Diabetes	0.01	7	—	0	2.2	—
Normal oral glucose tolerance test	—	36.8	0	—	36.8	0
Impaired oral glucose tolerance test	6.2	—	26.6	6.2	—	26.6
Diabetes	0.01	6.8	—	0.01	6.8	—
Normal HbA1c (at CVa = 3.5%)	—	48	1.3	—	16.5	0
Pre-diabetes	6.4	—	31.8	25.3	—	33.1
Diabetes	0.03	4.4	—	0	1.6	—
Normal HbA1c (at CVa = 2.0%)	—	30.5	0	—	11.6	0
Pre-diabetes	5.2	—	17.9	19.5	—	18.2
Diabetes	0	3.6	—	0	1.2	—

This is calculated by using the dividing the number of misclassified subjects with the original number of subjects within each diagnostic category.

When the laboratory tests for diabetes were repeated more than once, they improved the consistency of the disease classification over just using a single testing episode (Table 5). The average of the results of three repeat testing generally had better performance over the average results of two repeat testing or the ‘two of three’ classification strategy. The notable exception to this was the classification of prediabetes using HbA1c, which was most consistently made by the ‘two of three’ classification strategy. When the ‘two of three’ strategy was used, there were rare occasions when the three repeated results were all classified differently (i.e. no concordant results).

**Table 5:** Proportion of patients who are misclassified when laboratory testing for diabetes is repeated three times under different classification strategy.

**Tests**	**True classification**	**Classification after repeated tests**	**Classification strategy (World Health Organisation criteria)**				**Classification strategy (American Diabetes Association criteria)**			
			**Single test**	**Average of 2 repeats (%)**	**Average of 3 repeats (%)**	**2 out of 3 repeats (%)**	**Single test**	**Average of 2 repeats (%)**	**Average of 3 repeats (%)**	**2 out of 3 repeats (%)**
Fasting Plasma Glucose	Normal	Normal	96.8	98.4	99	98.4	91.5	94.6	96.1	95.3
		Impaired	3.2	1.6	1	1.6	8.5	5.4	3.9	4.7
		Diabetes	0	0	0	0 *(1 missing)*	0	0	0	0
	Impaired	Normal	27.2	18.6	19.1	18.1	25.3	19.8	21.4	20.5
		Impaired	63	77.9	76.5	71.6	71.6	77.4	77.4	77.8
		Diabetes	9.8	3.5	4.4	5.4 *(10 missing, 4.9)*	3.1	2.7	1.2	1.5 *(1 missing, 0.2)*
	Diabetes	Normal	0.3	0	0	0	0	0	0	0
		Impaired	10.2	6.5	7.2	6.5	10.5	6.5	7.2	7.2
		Diabetes	89.5	93.5	92.8	92.8 *(1 missing, 0.7)*	89.5	93.5	92.8	92.8
Oral glucose tolerance test	Normal	Normal	91.5	94.8	95.5	94.3	91.5	94.8	95.5	94.3
		Impaired	8.5	5.2	4.5	5.7	8.5	5.2	5.2	5.7
		Diabetes	0	0	0	0 *(1 missing)*	0	0	0	0 *(1 missing)*
	Impaired	Normal	26.9	19	17	18.3	26.9	19	17	18.3
		Impaired	61.7	73	76.6	68	61.7	73	76.6	68
		Diabetes	11.4	8	6.4	7.3 *(37 missing, 6.4)*	11.4	8	6.4	7.3 *(37 missing, 6.4)*
	Diabetes	Normal	1	0.4	0	0	1	0.4	0	0
		Impaired	15.9	13.3	9.7	8.5	15.9	13.3	9.7	8.5
		Diabetes	83.1	86.3	90.3	89.9 *(4 missing, 1.6)*	83.1	86.3	90.3	89.9 *(4 missing, 1.6)*
HbA1c	Normal	Normal	89.2	93	94.1	93.1	81.3	86.1	88.1	86.9
		Pre-diabetes	10.7	7	5.9	6.7	18.7	13.9	11.9	13.1
		Diabetes	0.1	0	0	0 *(6 missing, 0.2)*	0	0	0	0
	Pre-diabetesDiabetes	Normal	28.8	20.9	18.4	19.3	22.3	18.2	14.5	16.2
		Pre-diabetes	60	72.8	76.1	66.8	73.7	79.2	84	80.8
		Diabetes	11.2	6.3	5.5	6.1 *(44 missing, 7.7)*	4	2.6	1.5	2.6 *(7 missing, 0.4)*
		Normal	0.4	0	0	0	0	0	0	0
		Pre-diabetes	12.5	8	8	6.5	12.9	8	8	7.5
		Diabetes	87.1	92	92	92.5 *(2 missing, 1)*	87.1	92	92	92.5

## Discussion

This study provided an additional dimension to the discussion on the choice of laboratory test for identifying patients with glycaemic disorders in the community setting. The impact of biological and analytical variation on diabetes classification is related to the distribution of the laboratory results in the population examined as well as the diagnostic thresholds applied.

For example, for the diagnosis of prediabetes/impaired glucose tests, the classifications for the different tests under the WHO diagnostic criteria were relatively comparable. This was because the ratio of the diagnostic interval, defined as (upper limit of diagnostic threshold – lower limit of diagnostic threshold/lower diagnostic threshold) to the combined biological and analytical variation was comparable between the three tests (Table 6).

**Table 6:** Ratio of diagnostic interval to the combined biological and analytical variation for World Health Organisation (WHO) and American Diabetes Association (ADA) diagnostic criteria.

Diagnostic criteria		Diagnostic thresholds	Diagnostic interval (%)	Combined variation	Ratio of diagnostic interval to combined variation
WHO	FPG	6.1–6.9	13.1	6.2	2.1
	OGTT	7.8–11	41.0	16.9	2.4
	HbA1c	6–6.4	6.7	3.9	1.7
ADA	FPG	5.6–6.9	23.2	6.2	3.7
	OGTT	7.8–11	29.1	16.9	1.7
	HbA1c	5.7–6.4	12.3	3.9	3.1

FPG = fasting plasma glucose, OGTT = oral glucose tolerance test, HbA1c = glycated haemoglobin A1c. Diagnostic interval is calculated by [(upper threshold − lower threshold)/lower threshold], e.g. for FPG = [(6.9–6.1)/6.1 × 100].

On the other hand, when the ADA criteria were applied, the diagnostic intervals were widened for FPG and HbA1c while the OGTT remained unchanged. Hence, the ratios between the diagnostic interval to the combined variation for FPG and HbA1c were close to each other. This was reflected by the relatively comparable consistency in classification of subjects with prediabetes. By contrast, the OGTT had significantly lower ratio of diagnostic interval to combined variation, which was accompanied by much lower consistency in classifying subjects with prediabetes.

From the results of this study, HbA1c has comparable performance to FPG and is better than OGTT in classifying subjects with diabetes, particularly when laboratory methods with smaller CVa are used. Interestingly, some groups propose the testing strategy where a positive HbA1c test near the diagnostic threshold should be confirmed by OGTT, which is more likely to classify a patient erroneously.

For laboratory tests with small CVi, the CVa should be equally small so as not to increase the overall variability (noise) in the results. As a general rule of thumb, a CVa to CVi ratio of 0.75 is considered the minimum analytical requirement and a ratio of 0.25 is optimal^8^. At the former CVa specification, the analytical imprecision will add 25% of variability to the overall test result variability while the latter will add 3% variability. Because of the very tight CVi for HbA1c, the routine laboratory methods are unable to meet the stringent analytical requirement. Nevertheless, as shown in this work, choosing a HbA1c laboratory method with smaller CVa that is currently routinely available can improve the diagnostic performance considerably. Alternatively, CVa can be reduced by repeat testing on the same blood sample^5^.

Nonetheless, for single testing episodes, impact of the misclassification due to biological and analytical variations alone is perhaps better measured by the potential increase in disease prevalence. When assessed in this manner, the impaired glycaemia/ prediabetes category is most vulnerable to such misclassification, followed by diabetes. This can potentially have significant implication for epidemiological studies and classification of diabetes using FPG appears to be least affected by the random variations. By contrast, the presence of between-laboratory bias has different effect on different tests under different diagnostic criteria. In general, the ADA diagnostic criteria are more resilient than the WHO diagnostic criteria to the effect of between-laboratory bias.

This study also sought to resolve the conundrum of interpreting the results of repeat laboratory tests. The use of the average of the results of the repeat laboratory tests has the effect of ameliorating the combined (analytical and biological) variation. The averaged result improves the consistency of the disease classification.

In theory, HbA1c should be the test that will most consistently classify subjects with diabetes given its narrow CVi and CVa (i.e. lowest total variability). However, another important factor in that may affect the diagnostic performance of a qualitative test is the distribution of data around the diagnostic thresholds. Once this was considered, FPG performed better than HbA1c. Because the performance of these biomarkers is dependent on the laboratory performance and the population examined, including different age and ethnic groups that may show significantly different result distribution^9^, they should be verified with local data to optimise decision making.

## Electronic supplementary material

Supplemental Data
