# Supplementary material for: Impact of analytical and biological variations on classification of diabetes using fasting plasma glucose, oral glucose tolerance test and HbA1c
Source: Sci Rep. 2017 Oct 20;7:13721. doi: 10.1038/s41598-017-14172-8 (PMC5651837; doi:10.1038/s41598-017-14172-8)

Title: Impact of analytical and biological variations on classification of diabetes using fasting plasma glucose, oral glucose tolerance test and HbA1c

Jia Hui Chai,^1^ Stefan Ma,^2^ Derick Heng,^3^ Joanne Yoong,^1^ Wei-Yen Lim,^4^ Sue-Anne Toh,^5^ Tze Ping Loh^6,7^*

Affiliations:

^1^Saw Swee Hock School of Public Health, National University of Singapore, Singapore

^2^Epidemiology & Disease Control Division, Ministry of Health, Singapore.

^3^Public Health Group, Ministry of Health, Singapore

^4^Research and Development Office, Agency for Integrated Care, Singapore

^5^Department of Medicine, National University Hospital, Singapore

^6^Department Laboratory Medicine, National University Hospital, Singapore

^7^Biomedical Institute for Global Health Research and Technology, National University of Singapore, Singapore

Supplemental Table 1. Correlation among the three laboratory tests in the study population. FPG = fasting plasma glucose, OGTT = oral glucose tolerance test, HbA1c = glycated haemoglobin A1c.

| Variables | Fasting plasma glucose | Oral glucose tolerance test | HbA1c |
| --- | --- | --- | --- |
| Fasting plasma glucose | 1 |  |  |
| Oral glucose tolerance test | 0.788 | 1 |  |
| HbA1c | 0.869 | 0.796 | 1 |

Supplemental Figure 1. Histogram showing the distribution of the fasting plasma glucose of the subjects without prior history of diabetes mellitus. The red vertical lines represent the diagnostic thresholds (WHO criteria) for impaired fasting glucose and diabetes, respectively. The 95% confidence intervals, as represented by two standard deviations of the combined biological variation (5.7%) and analytical variation (2.5%) of the diagnostic thresholds, are indicated with blue arrows.


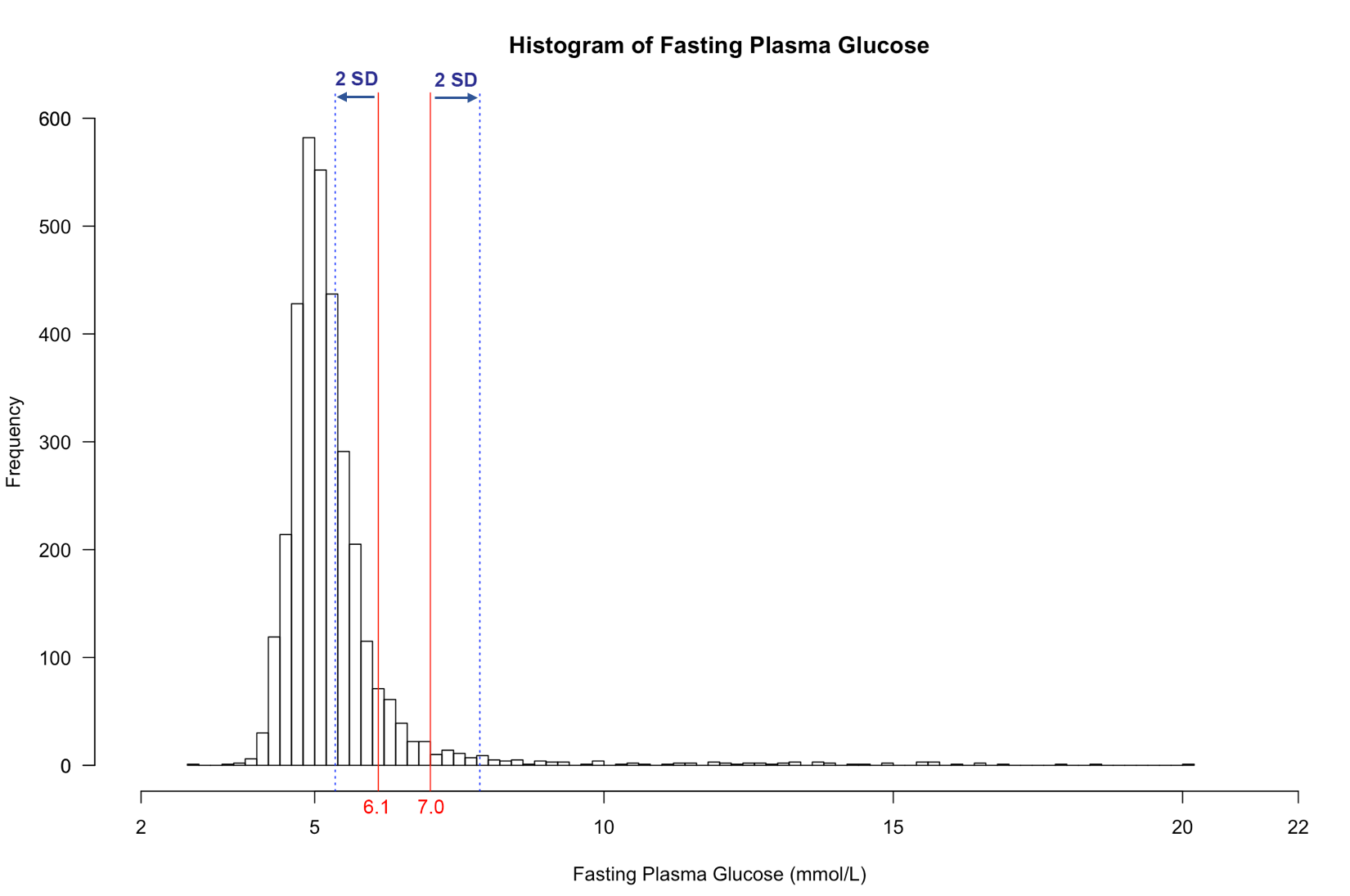


Supplemental Figure 2. Histogram showing the distribution of the oral glucose tolerance test of the subjects without prior history of diabetes mellitus. The red vertical lines represent the diagnostic thresholds (WHO criteria) for impaired glucose tolerance and diabetes, respectively. The 95% confidence intervals, as represented by two standard deviations of the combined biological variation (16.7%) and analytical variation (2.5%) of the diagnostic thresholds, are indicated with blue arrows.


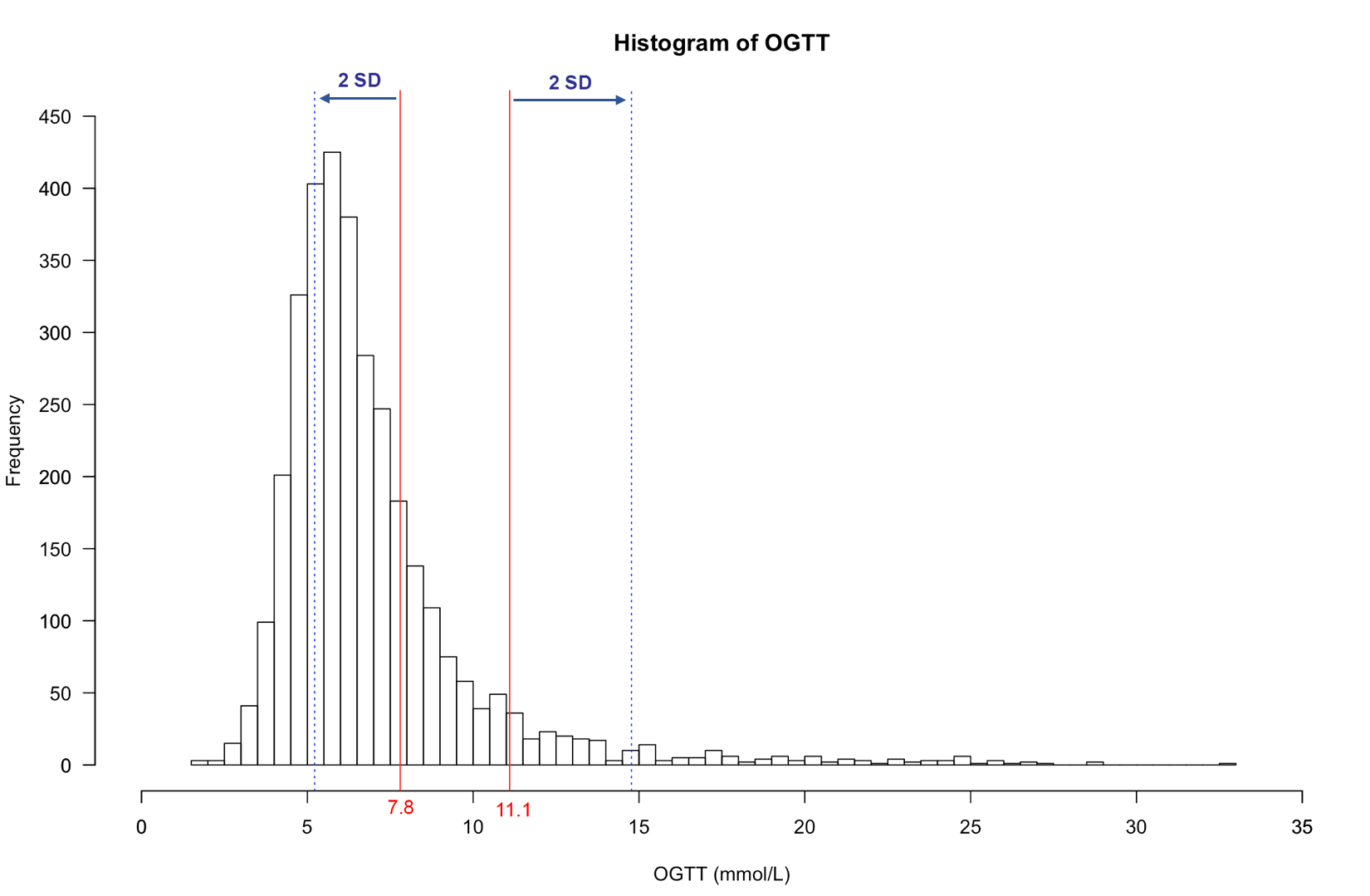


Supplemental Figure 3. Histogram showing the distribution of the HbA1c test of the subjects without prior history of diabetes mellitus. The red vertical lines represent the diagnostic thresholds (WHO criteria) for pre-diabetes and diabetes, respectively. The 95% confidence intervals, as represented by two standard deviations of the combined biological variation (1.8%) and analytical variation (3.5%) of the diagnostic thresholds, are indicated with blue arrows.


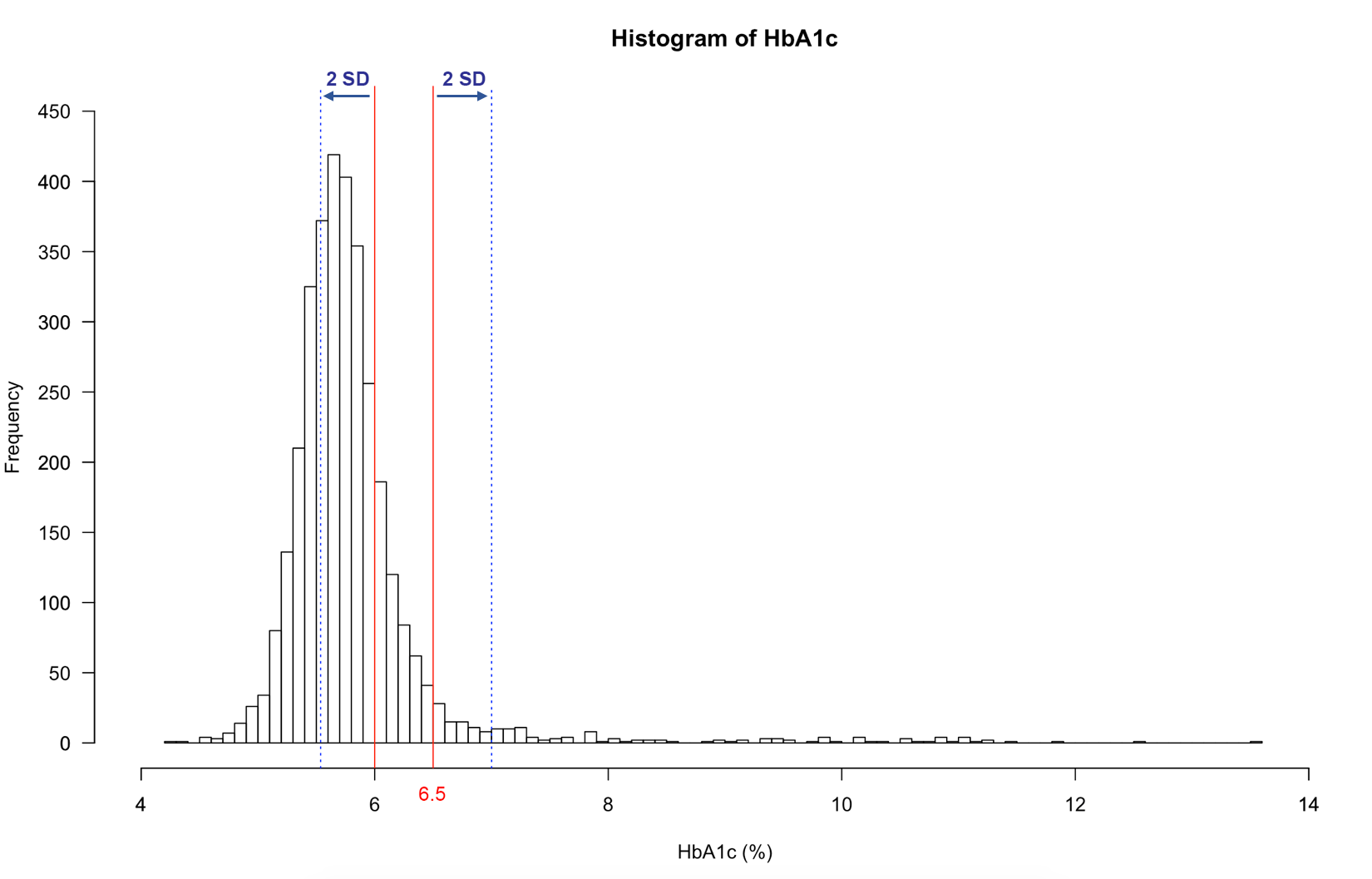


Supplemental Figure 4. Density plot of fasting plasma glucose results by race.


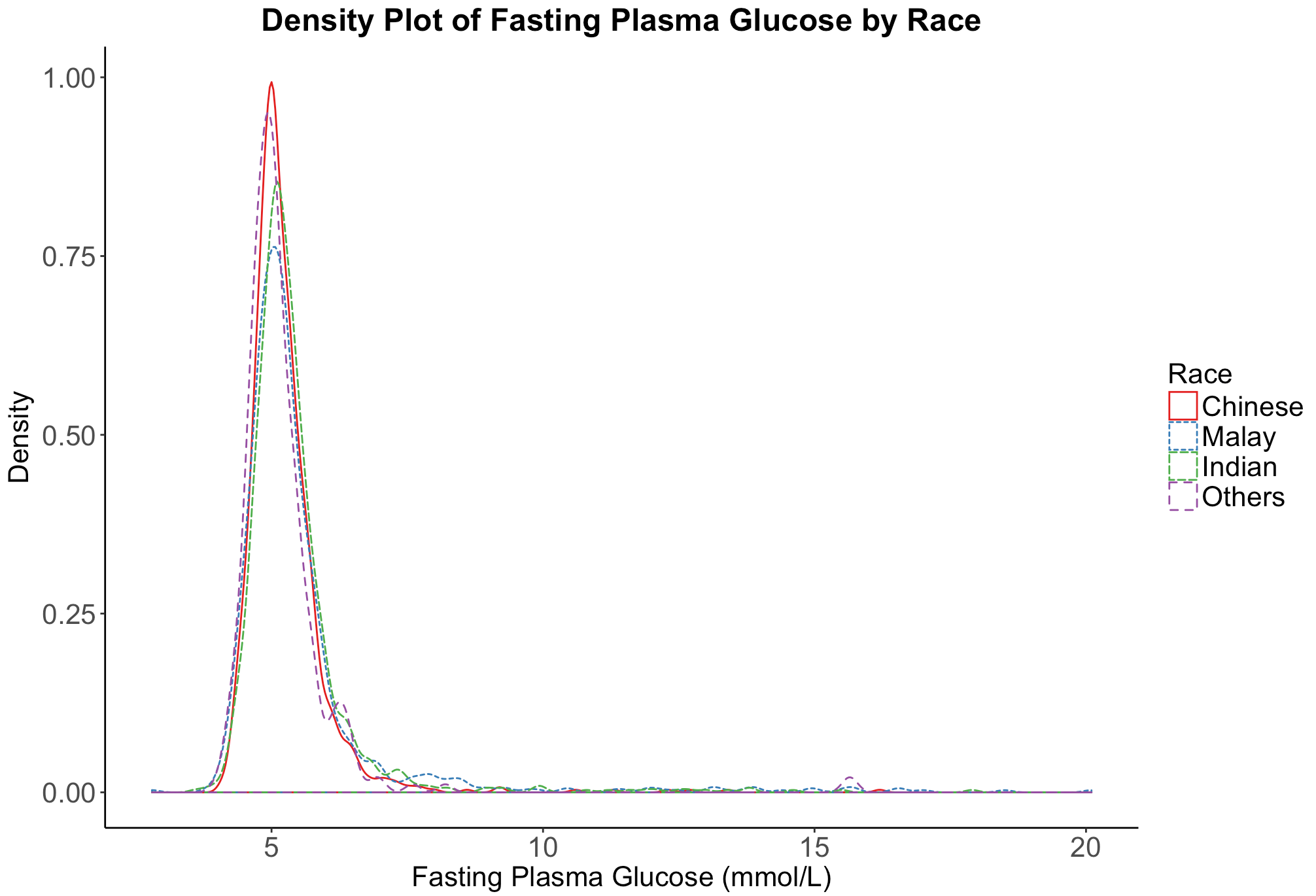


Supplemental Figure 5. Density plot of oral glucose tolerance test (OGTT) results by race.


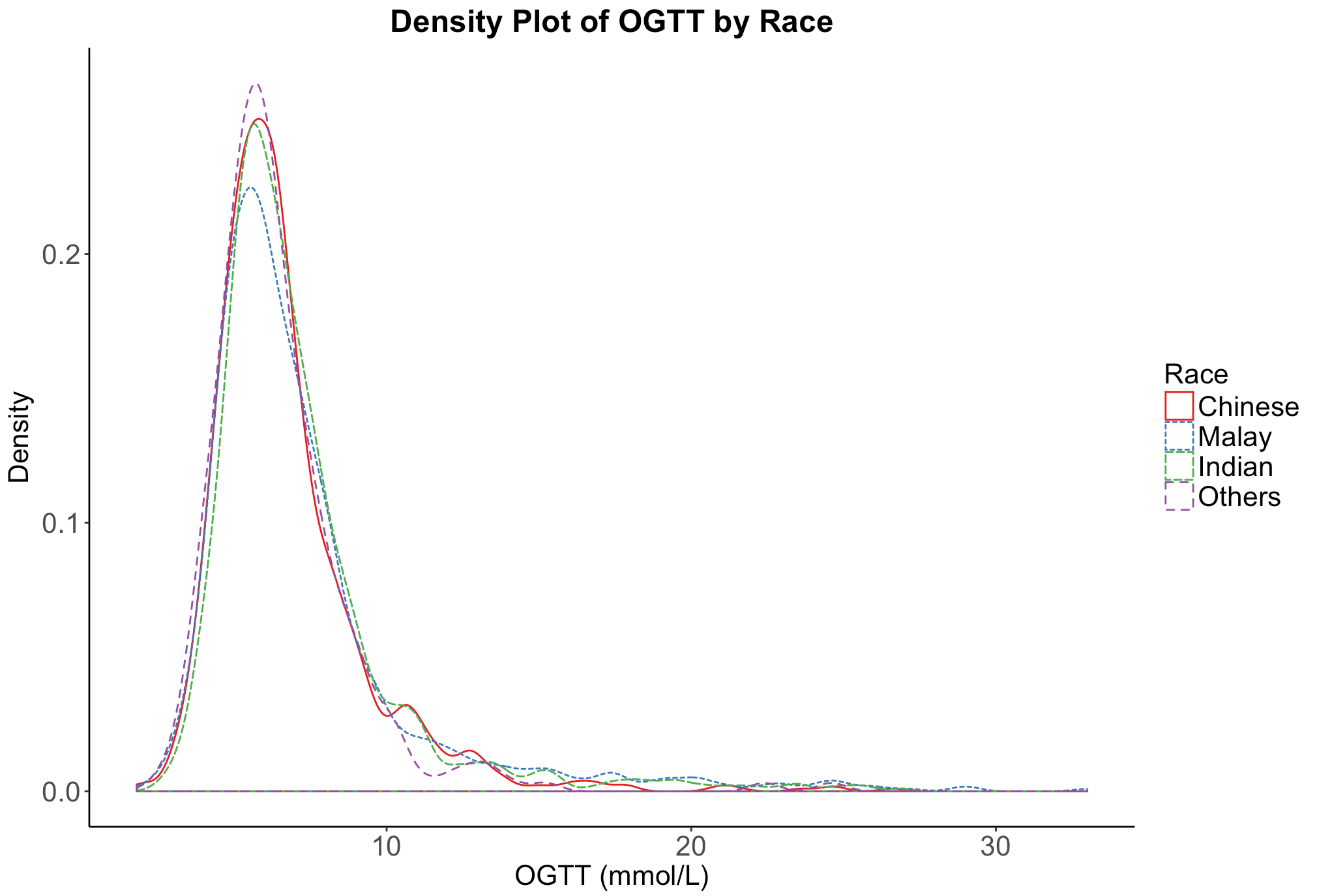


Supplemental Figure 6. Density plot of glycated haemoglobin A1c (HbA1c) results by race.


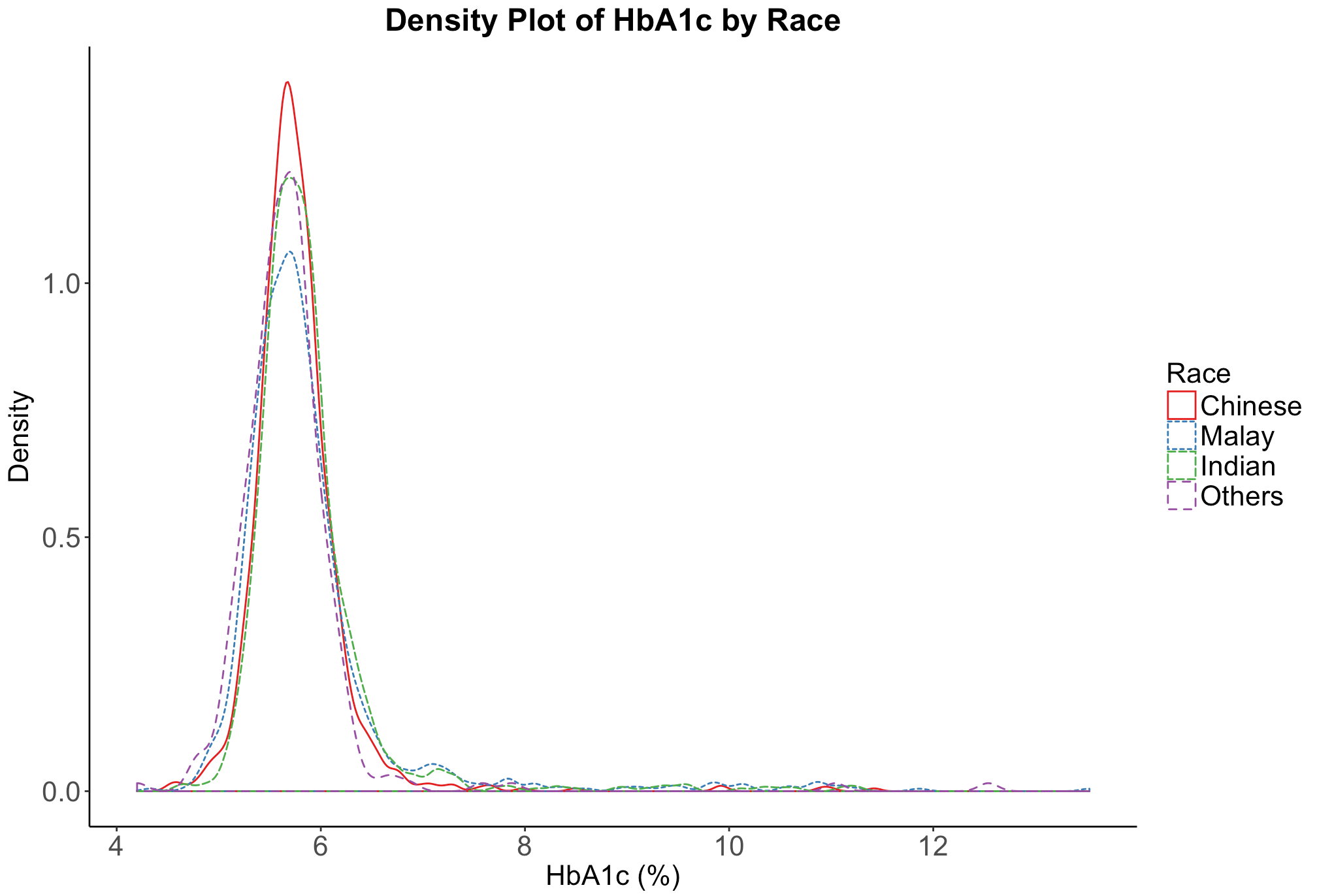

Supplement: Supplementary file 1 — Supplemental Data [file 41598_2017_14172_MOESM1_ESM.doc]
